# A scalable neural network emulator with MRAM-based mixed-signal circuits

**DOI:** 10.3389/fnins.2025.1599144

**Published:** 2025-06-09

**Authors:** Jua Lee, Jiho Song, Hyeon Seong Im, Jonghwi Kim, Woonjae Lee, Wooseok Yi, Soonwan Kwon, Byungsu Jung, Joohyoung Kim, Yoonmyung Lee, Jung-Hoon Chun

**Affiliations:** ^1^College of Information and Communication Engineering, Sungkyunkwan University (SKKU), Suwon, Republic of Korea; ^2^Memory Division, Samsung Electronics, Hwaseong, Republic of Korea; ^3^Samsung Advanced Institute of Technology (SAIT), Samsung Electronics, Suwon, Republic of Korea; ^4^IMEC, Leuven, Belgium

**Keywords:** analog neural network, biological neural network, refractory period, lateral inhibition, inhibitory post synaptic potential

## Abstract

In this study, we present a mixed-signal framework that utilizes MRAM (Magneto-resistive Random Access Memory) technology to emulate behaviors observed in biological neural networks on silicon substrates. While modern technology increasingly draws inspiration from biological neural networks, fully understanding these complex systems remains a significant challenge. Our framework integrates multi-bit MRAM synapse arrays and analog circuits to replicate essential neural functions, including Leaky Integrate and Fire (LIF) dynamics, Excitatory and Inhibitory Postsynaptic Potentials (EPSP and IPSP), the refractory period, and the lateral inhibition. A key challenge in using MRAM for neuromorphic systems is its low on/off resistance ratio, which limits the accuracy of current-mode analog computation. To overcome this, we introduce a current subtraction architecture that reliably generates multi-level synaptic currents based on MRAM states. This enables robust analog neural processing while preserving MRAM’s advantages, such as non-volatility and CMOS compatibility. The chip’s adjustable operating frequency allows it to replicate biologically realistic time scales as well as accelerate experimental processes. Experimental results from fabricated chips confirm the successful emulation of biologically inspired neural dynamics, demonstrating the feasibility of MRAM-based analog neuromorphic computation for real-time and scalable neural emulation.

## 1 Introduction

Neuromorphic circuits have been proposed as a promising path for advancing the next generation of computing technologies by drawing inspiration from the organizing principles of biological nervous systems (Basu et al., 2022; Indiveri and Horiuchi, 2011; Indiveri et al., 2011; Mead, 1990; Thakur et al., 2018; Yang et al., 2020). A wide range of neuromorphic architectures and large-scale emulation systems have been reviewed and compared in recent studies, highlighting their potential to efficiently replicate the computational capabilities of biological neural networks while addressing energy and scalability challenges (Thakur et al., 2018). These circuits not only emulate the computational capabilities of individual neurons but also employ spiking representations for communication, learning, memory, and computation. However, despite their reliance on biological neural networks as a reference, our understanding of these complex systems remains limited.

Research efforts have been actively directed toward capturing more detailed signals in biological neural networks. For instance, recent developments have introduced nano-electrode arrays (Abbott et al., 2020) capable of recording signals in biological neural networks. These arrays allow for the cultivation of neural networks directly on the surface of an integrated circuit, establishing connections with neurons. These developments motivate the need for hardware platforms capable of real-time interaction with biological signals, operating at biologically realistic time scales, and supporting biologically meaningful behaviors such as the refractory period and lateral inhibition.

Numerous prior studies have incorporated biological neural networks into circuits and systems. However, in most of these studies, synaptic weights were stored using SRAM (Akopyan et al., 2015; Benjamin et al., 2014; Bose et al., 2020; Chen et al., 2019; Davies et al., 2018; Merolla et al., 2011; Schemmel et al., 2010; Schemmel et al., 2010; Seo et al., 2011; Zhao et al., 2019) or memristor devices such as RRAM or PCM. (Burr et al., 2015; Chiu et al., 2023; Jo et al., 2010; Kulkarni et al., 2019; Ostwal et al., 2019; Park et al., 2013; Sun et al., 2018; Valentian et al., 2019; Verma et al., 2023; Vincent et al., 2015; You et al., 2023; Zamarreño-Ramos et al., 2011; Zhang et al., 2018), while computation was performed through digital methods (Painkras et al., 2013; Stuijt et al., 2021). Although several works have explored the use of MRAM (Chiu et al., 2023; Kulkarni et al., 2019; Ostwal et al., 2019; Verma et al., 2023; Vincent et al., 2015; You et al., 2023), they have predominantly focused on simulation-level evaluations. Based on the existing literature, there has been no prior demonstration of a fabricated neuromorphic system employing MRAM that incorporates biologically inspired neural architectures with analog membrane potential dynamics. In this work, we explore the use of MRAM for implementing synaptic weights due to its non-volatility, CMOS compatibility, and process maturity (Basu et al., 2018; Na et al., 2020). While MRAM has notable benefits, it presents a key challenge for use in biologically plausible analog systems: its low on/off resistance ratio, which limits accurate current-mode analog computation within mixed-signal architectures. The proposed architecture introduces a current subtraction method that compensates for MRAM’s limited resistance contrast and enables reliable multi-level current generation. Specifically, the configurable timing via global clock (GCLK) controlling synaptic integration and AXON input timing can be set between 1 kHz and 50 kHz, covering biological firing rates (e.g., 1–100 Hz) as well as accelerated conditions for faster experimentation. Furthermore, the architecture is implemented in a modular local cluster format, consisting of MRAM synapse arrays, analog neuron circuits, and interface blocks. While this paper focuses on a single-cluster implementation, multiple clusters can be tiled together using packet-based routing (TX/RX/Router), enabling scalability toward a complete, large-scale system.

The rest of this paper is organized as follows. Section 2.1 presents the comprehensive architecture featuring MRAM circuits and details the types of neural behaviors from biological neural networks that the proposed design can emulate. Section 2.2 describes the design and implementation of the proposed mixed-signal architecture. Section 3 covers the measurement procedures and results. Finally, Section 4 provides a summary of the findings.

## 2 Materials and methods

### 2.1 Neural network basics and a comprehensive architecture with MRAM

#### 2.1.1 Overall architecture

**FIGURE 1:**
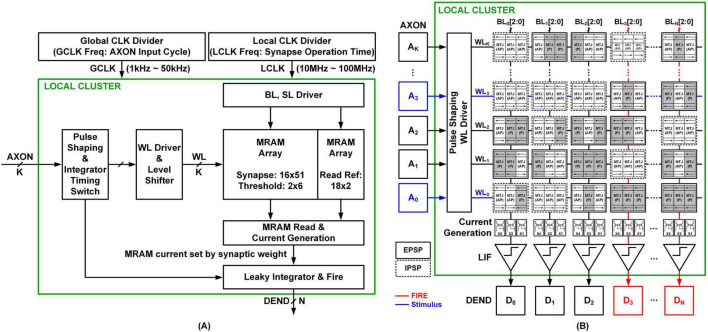
Cluster design for mixed-signal neural emulation. **(A)** Single cluster architecture with K axons and N dendrites. **(B)** MRAM array architecture.

Figure 1A provides an overview of the comprehensive architecture for mixed-signal neural emulation, which is primarily composed of three key elements: the memory array, the memory read and current generation circuit, and the integrator circuit. The memory array consists of threshold weights and synaptic weights, with each synaptic weight represented by three bits. The synapse bit-width (currently 3 bits) is flexible and can be adjusted depending on system requirements; however, such changes would require corresponding modifications to the peripheral control logic. The memory read and current generation circuit, detailed in section 2.1.3, plays a crucial role in this architecture. The architecture represents a local neuron cluster comprising K AXONs and N DENDs, where K = N = 16 in the implemented chip. K and N denote the network’s input and output scalability dimensions, respectively. In Figure 1, only the WL and BL are shown for simplicity, providing a high-level overview of the crossbar architecture. Each cluster includes 16 × 51 MRAM cells: 16 × 48 cells for storing actual synaptic weights and 16 × 3 cells for PSP reference circuits, supporting current-mode differentiation. Further details are provided in Figure 5B. In addition, the 2 × 6 MRAM cells are allocated for threshold storage, and 18 × 2 MRAM cells are used as read references to facilitate reliable state sensing. Multiple local clusters can be tiled together using interface circuits to scale up the network size. The global clock (GCLK) governs the axon input cycle, while the local clock (LCLK) controls the synapse operations.

When an AXON spike arrives in synchronization with GCLK at the local cluster, the pulse shaping logic activates internal switches using LCLK to control the memory array and integrator circuit. The spike input is converted to a word line (WL) input by the pulse shaping logic and WL drivers, enabling the control of the memory array through bit line (BL) and source line (SL) drivers. While input timing in biological neural networks is inherently unsynchronized, circuit designs often assume specific input frequencies to ensure proper functionality. The integrator timing switch logic activates several key switches, including reset, integration time, comparator, and leakage switches, as detailed in section 2.2.1. The current generated by the memory read and current generation circuit-determined by the synaptic weight-is subsequently passed to the integrator. Depending on the resulting current, the DEND may fire a spike, emulating the action potential generation process in biological neurons.

Figure 1B elucidates the structure of the memory array, effectively representing the synapse. The WL of the memory receives the transmitted stimuli from the axon, and the column’s output transmits the resulting information to the dendrite. Each synaptic weight consists of a 3-bit memory that can be programmed individually. The current generation circuit applies different current mirror ratios (e.g., x1, x2, or x4) to set the weights in binary form. Depending on the programmed MRAM values, the synapse can implement either an Excitatory Postsynaptic Potential (EPSP) or an Inhibitory Postsynaptic Potential (IPSP). This EPSP/IPSP distinction is visually represented in the figure using solid- or dashed-line boxes, and it applies exclusively to the MRAM synaptic weights. No such distinction is made for AXONs or DENDs, as these components in the figure represent only the signal flow path—AXONs deliver input spikes, and DENDs receive integrated current and generate output spikes. Each column shares its current generation circuits, allowing simultaneous inputs to be integrated within the column. This shared structure emulates biological spatial summation by summing the incoming currents at the dendritic side.

In addition to synaptic weights, the memory array also includes threshold weights, which determine the neuron’s firing threshold by setting the duration of the integration window. These threshold values are stored in dedicated MRAM cells—six MRAM devices per cluster—and are shared by all neurons within the local cluster. This shared threshold reference is used by the integrator timing switch circuit to adjust the integration time window and ensure consistent integration behavior across neurons. While more granular threshold control (e.g., per-column or per-neuron) is possible, it was not implemented in this work to maintain area efficiency and circuit simplicity.

#### 2.1.2 Biological neural networks

**FIGURE 2:**
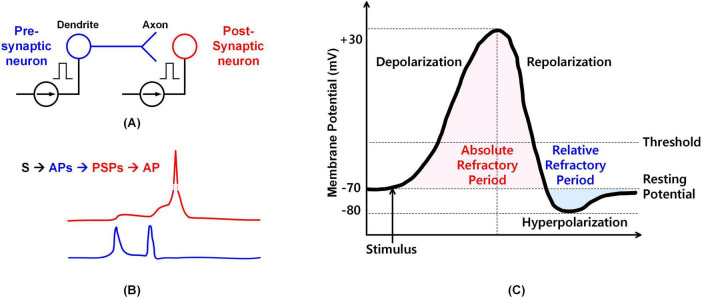
Neuron transmission and membrane potential. **(A)** Stimulus transmission between pre-neuron and post-neuron. **(B)** Membrane potential of a real biological neuron during stimulus transmission. **(C)** Membrane potential corresponding to different neuron states.

Neurons transmit information through synapses, where electrical impulses travel from the axon of a pre-synaptic neuron to the dendrite of a post-synaptic neuron, as illustrated in Figure 2A (Abbott et al., 2020). Figure 2B shows the recorded membrane potential of cultured neurons from (Abbott et al., 2020), demonstrating that when a pre-synaptic neuron generates a spike, it propagates across the synapse, causing an increase in the post-synaptic neuron’s membrane potential. In this context, “S” denotes a stimulus, “AP” represents an action potential, and “PSP” stands for post-synaptic potential. When the membrane potential exceeds a defined threshold, the post-synaptic neuron fires a spike and then returns to its resting potential.

The Leaky Integrate-and-Fire (LIF) model abstracts this neural behavior into three key processes: input integration, leakage, and firing/reset. Neurons integrate incoming stimuli, with the PSP reflecting changes in membrane potential. The “leaky” property refers to the gradual decay of membrane potential over time in the absence of new input, representing the neuron’s tendency to return to its resting state. When the integrated membrane potential surpasses the threshold, the neuron fires and resets.

Figure 2C illustrates membrane potential variations at different stages (Yang et al., 2020). In the resting state (approximately –70 mV), the neuron is polarized. A stimulus induces depolarization, and if the potential exceeds the threshold, an action potential is triggered, followed by repolarization and, occasionally, hyperpolarization before returning to the resting state.

Post-synaptic potentials (PSPs) can be either excitatory (EPSP) or inhibitory (IPSP), depending on the nature of the stimulus. The likelihood of generating an action potential depends on whether the combined effect of stimuli causes a sufficient increase in membrane potential. Multiple EPSPs and IPSPs can summate spatially or temporally to influence the neuron’s firing behavior.

While the LIF model captures fundamental neural dynamics, real neurons exhibit additional complexities, including the refractory period and lateral inhibition. The refractory period, depicted in Figure 2C, consists of an absolute phase—during which the neuron is unresponsive to stimuli—and a relative phase, which requires a stronger-than-usual stimulus to elicit a response. Lateral inhibition, commonly observed in visual neurons, prevents adjacent neurons from firing immediately after one has fired. This mechanism plays a critical role in visual processing and is exemplified by phenomena such as the Hermann grid illusion.

#### 2.1.3 MRAM as synapse array

In this study, MRAM is chosen to represent synaptic weights due to its non-volatile nature. These characteristics ensure the retention of synaptic weight information, particularly in experiments that replicate the operations of biological neural networks, as demonstrated in Abbott et al. (2020), or in scenarios involving learning capabilities. Furthermore, MRAM provides advantages such as fast data processing, low power consumption, and easy integration, owing to its compact size (Basu et al., 2018; Na et al., 2020).

##### 2.1.3.1 Characteristics of MRAM

**FIGURE 3:**
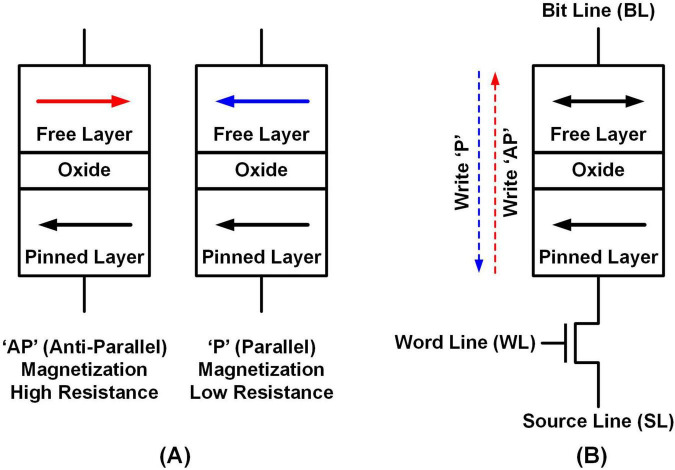
**(A)** MRAM configuration: AP state and P state, **(B)** MRAM write operation.

MRAM utilizes a magnetic tunneling junction (MTJ), which consists of a pinned layer and a free layer made of ferromagnetic material, separated by an insulating oxide layer. As depicted in Figure 3A, the resistance of MTJ is determined by the direction of current passing through it, resulting in two distinct resistance states: the low-resistance parallel state (LRS, P) and the high-resistance anti-parallel state (HRS, AP) (Na et al., 2020).

Figure 3B illustrates the 1T1M MRAM configuration, including one access transistor and one MTJ. The access transistor is connected to the pinned layer of the MTJ, with its source connected to the MRAM’s source line (SL) and its gate connected to the word line (WL). Meanwhile, the free layer is linked to the bit line (BL) of the MRAM. To write data into the MRAM cell, the current flows either from the pinned layer to the free layer to achieve the AP state or from the free layer to the pinned layer to achieve the P state.

##### 2.1.3.2 MRAM read circuit

**FIGURE 4:**
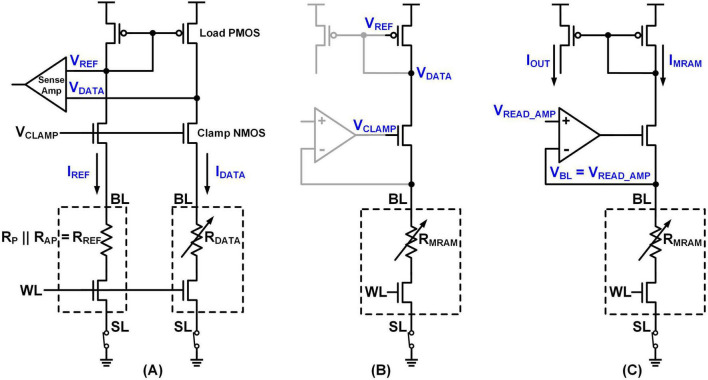
**(A)** Conventional MRAM read circuit and proposed MRAM read circuit **(B)** in memory read operation and **(C)** in synapse operation.

The conventional MRAM read circuit (Na et al., 2020), depicted in Figure 4A, employs a Clamp NMOS to generate currents (I_*DATA*_, I_*REF*_) proportional to the resistance value of the MTJ. Theses currents are then converted into voltages (V_*DATA*_, V_*REF*_) using a Load PMOS. By comparing I_*DATA*_ with I_*REF*_ or V_*DATA*_ with V_*REF*_, the MRAM state (P or AP) can be identified. The MRAM devices are connected between the BL and the SL, with the SL tied to GND. Selection of each MRAM device is controlled by the WL.

To enable both memory read (distinguishing between P and AP states) and synapse operations (current generation for an integrator), we propose a dual-mode read circuit shown in Figures 4B,C. For memory read operations (Figure 4B), voltage comparison is performed while the regulation amplifier remains off. In contrast, during synapse operations (Figure 4C), the amplifier is activated, fixing V_*BL*_ at V_*READ_AMP*_. The diagrams clearly show the connections of BL, WL, and SL, as well as the readout current path from the MRAM and to the integrator. To prevent MRAM read disturbance (Na et al., 2020), V_*READ_AMP*_ must be maintained at a very low level, ideally below 50 mV. The resulting output current, determined by the resistance value, is then fed into the integrator, which will be elaborated on in section 2.2.

Regarding array tiling, the memory read circuit and synapse operation current generation circuit are shared across each column of the crossbar array. Specifically, when the BL and SL of the MRAM devices are connected, the corresponding synapses within that column utilize a shared readout and current integration circuit. Each column has a dedicated read path, while the WLs are used to select specific rows during memory operations.

#### 2.1.4 Proposed MRAM architecture

**FIGURE 5:**
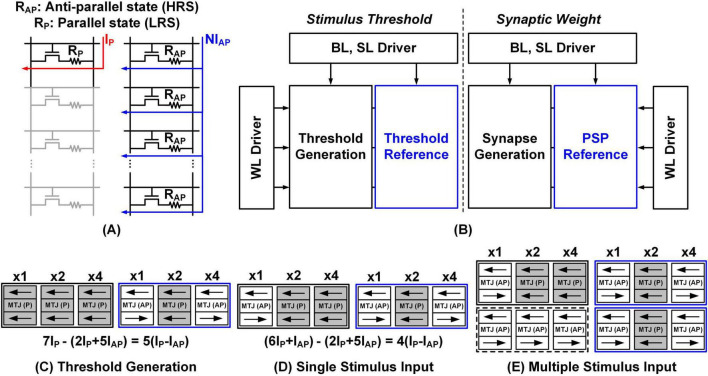
**(A)** Limitation caused by low TMR ratio. **(B)** Proposed MRAM architecture for threshold and synapse current generation. Examples of **(C)** threshold current generation, **(D)** single stimulus input, and **(E)** multiple simultaneous stimuli.

In the MRAM used in this study, the resistance ratio between the P state and the AP state is relatively small, ranging from 10 kΩ:20 kΩ to 5 kΩ:30 kΩ at most. When the resistance ratio is 1:2, it becomes challenging to distinguish a single P state MRAM cell from two AP state MRAM cells when two WLs are accessed simultaneously to read the data. This issue, illustrated in Figure 5A highlights the difficulty of distinguishing a single P state from multiple AP states. Here, I_*P*_ and I_*AP*_ are currents flowing through single P-state and AP-state MRAM cells, respectively. For example, if I_*P*_/I_*AP*_ is 2, we cannot differentiate I_*P*_ and 2xI_*AP*_. The problem can persist even with other resistive memories featuring a higher on/off ratio, particularly as the array size increases. To resolve this, we propose a method for subtracting the current contribution from AP states.

We use multi-bit representations for all synaptic weights and thresholds to enhance precision and flexibility in neuron modeling. Figure 5B shows the memory architecture for generating currents with multi-bit components. Word lines (WLs) connect the threshold generation and its reference blocks, mirroring a similar configuration for the synapse generation and PSP reference blocks. Each memory unit is independently accessible and programmable. The comparison of synapse generation bits with PSP reference bits determines the type of post-synaptic potential: if the synapse generation bits exceed the PSP reference bits, an EPSP is indicated; otherwise, an IPSP is represented. Figure 5C elaborates on the concept of threshold current generation. For instance, if the threshold generation block contains seven P-state cells and the threshold reference block consists of two P-state and five AP-state cells, the resulting threshold current is calculated as the difference between the two, yielding 5(I_*P*_ –I_*AP*_) or 5α, where α is defined as I_*P*_ –I_*AP*_. Similarly, in Figure 5D, for a single stimulus input, the PSP reference block consists of two P-state and five AP-state cells, while the synapse generation block includes six P-state and one AP-state cells. This configuration corresponds to an EPSP with a net current calculated as 4(I_*P*_-I_*AP*_) or 4α. If the threshold is set to 5α as shown in Figure 5C, and a stimulus of 4α is applied, as shown in Figure 5D, the stimulus exceeds the threshold, thereby triggering an action potential.

The same principle applies when multiple stimulus inputs are present. Figure 5E illustrates the PSP reference and two simultaneous stimulus blocks. The first row of the synapse generation block includes six P-state and one AP-state cells, representing an EPSP of 4α. The second row of the synapse generation block, which contains seven AP-state cells, represents an IPSP of –2α. When these two stimuli are applied simultaneously, their effects are summated, resulting in a net current of 2α. However, since this net current remains below the threshold of 5α, defined in Figure 5C, no action potential is triggered.

### 2.2 Analog circuit implementation

#### 2.2.1 Integrator

##### 2.2.1.1 Basic architecture

**FIGURE 6:**
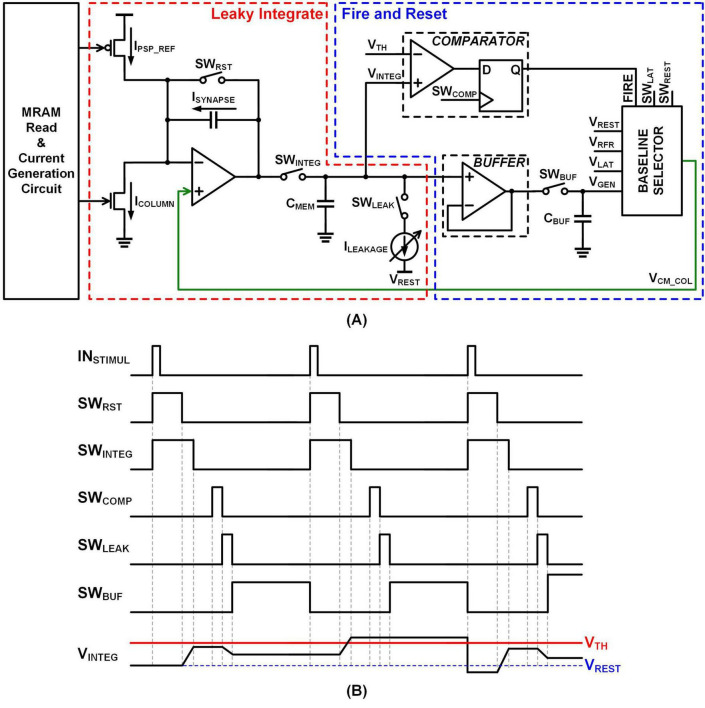
Leaky integrate and fire **(A)** schematic **(B)** timing diagram.

Figure 6A illustrates a circuit designed to emulate the LIF functionality of a biological neural network, serving as a key component of the system shown in Figure 1A. This circuit processes stimuli, corresponding to inputs received via WLs. I_*PSP_REF*_ is obtained from the PSP reference array block shown in Figure 5B, while I_*COLUMN*_ is generated by the Synapse Generation array block in the same figure. As described in section 2.1.4, the synaptic input current, I_*SYNAPSE*_ is computed as the difference between the synaptic weight and the PSP reference, specifically I_*SYNAPSE*_ = I_*COLUMN*_ – I_*PSP_REF*_. This indicates that the input current to the integrator is determined by the synaptic weight. This LIF circuit utilizes the current output from the memory array as its input and produces an action potential (FIRE) as an output signal. Figure 6B presents a timing graph depicting the operation of the circuit, particularly the timing of the integrator’s switches. All signals, except for the SW_*INTEG*_ signal, are generated using edge-detection techniques implemented with D flip-flops driven by GCLK and LCLK.

In actual biological neural networks, the timing of inputs and neural activity does not adhere to a fixed or synchronized frequency. However, when designing circuits to emulate the functionality of biological neural networks, it is typically assumed that inputs are received at a specific frequency. To facilitate circuit operation, signals are adjusted and reconstructed to align with this assumed frequency, as shown in Figure 1A through Pulse Shaping & Integrator Timing Switch.

Circuits designed to mimic the functionality of biological neural networks can be categorized into two operations: Leaky Integrate (indicated in red in Figure 6A) and Fire and Reset (indicated in blue in Figure 6B) operations. The currents described in section 2.1.3 are applied to the integrator input in Figure 6A, where the difference between the currents is converted into a voltage. When SW_*RST*_ and SW_*INTEG*_ are on, the input and output voltages of the integrator amplifier are set to V_*CM_COL*_, and this voltage is stored in the capacitor. After SW_*RST*_ is deactivated while the SW_*INTEG*_ remains active, the voltage begins to change based on the value of I_*SYNAPSE*_. In biological systems, leakage occurs naturally; in Figure 6A, this leakage is emulated using the I_*LEAKAGE*_ current when the SW_*LEAK*_ is connected. SW_*LEAK*_ is turned on after the threshold comparison operation, causing leakage toward V_*REST*_. If V_*INTEG*_ > V_*REST*_, the voltage decreases, and if V_*INTEG*_ < V_*REST*_, the voltage increases. The degree of leakage can be adjusted by controlling the pulse width of SW_*LEAK*_ and the magnitude of the I_*LEAKAGE*_. A comparator is used to compare V_*INTEG*_ with V_*TH*_. Base on the SW_*COMP*_ signal, the comparator generates a FIRE signal (an action potential) when the threshold condition is met.

To represent the relative refractory period and lateral inhibition, as discussed in section 2.1.2, we used a baseline selector. This component defines the voltage levels corresponding to various states: V_*GEN*_ for the leakage state without action potential emission, typically in response to subthreshold stimuli; V_*RFR*_ for the relative refractory period state; V_*REST*_ for the resting state; and V_*LAT*_ for the state associated with lateral inhibition. The voltage levels are configured to satisfy the following relationship: V_*LAT*_ < V_*RFR*_ < V_*REST*_. Each voltage level can be individually adjusted to suit specific requirements. During the initial integration phase, the baseline selector’s output voltage is set to V_*REST*_, controlled by SW_*REST*_. If a stimulus raises the voltage without triggering an action potential, the baseline selector’s output transitions to V_*GEN*_, when all selection bits are set to 0.

In contrast, the absolute refractory period is enforced through the operation of the switch generation block, which is governed by the GCLK. Once an input stimulus triggers an action potential, the switch generator becomes inactive for a fixed interval, during which no new switches are generated – regardless of subsequent inputs – thereby ensuring that the neuron remains entirely unresponsive during this period.

The baseline selector and switch generation block define the neuron’s behavior across various physiological states. The output voltage of the baseline selector determines the starting voltage of the integrator for processing subsequent stimuli, thereby influencing the overall neuronal response dynamics.

##### 2.2.1.2 Time window generation

**FIGURE 7:**
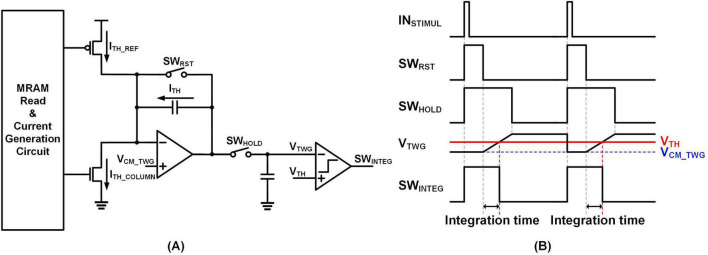
Time window generator. **(A)** Circuit schematic. **(B)** Timing diagram.

Figure 7A depicts a circuit producing the SW_*INTEG*_ signal, which is subsequently applied to the leaky integrate circuit shown in Figure 6, utilizing the same integrator structure. Current inputs from the MRAM represent the stimulus threshold. When an input stimulus arrives, SW_*RST*_ and SW_*HOLD*_ are connected, resetting the amplifier’s common mode voltage to V_*CM_TWG*_. In this configuration, V_*CM_TWG*_ is set to a voltage lower than the integrator’s actual starting voltage. This arrangement ensures the generation of an action potential when the MRAM weight values for the threshold and synaptic weights are identical.

After the input stimulus is received, with SW_*RST*_ on and SW_*HOLD*_ off, V_*TWG*_ increases as a result of the difference I_*TH_COLUMN*_ – I_*TH_REF*_. V_*TWG*_ connects to a comparator via an analog amplifier. If V_*TWG*_ surpasses V_*TH*_, SW_*INTEG*_ is set to 0. SW_*INTEG*_ signal is sent to the circuit in Figure 6, where it determines integration time from the falling edge of SW_*RST*_ to the falling edge of SW_*INTEG*_.

In the current implementation, a single threshold reference is shared across all neurons within a local cluster to achieve efficient hardware utilization. Although finer granularity could be achieved by assigning a unique threshold per column or per neuron, such approaches would significantly increase area and circuit complexity. Therefore, the single-threshold-per-cluster strategy is adopted as a practical trade-off between scalability, flexibility, and area efficiency.

On the test chip, the generation of SW_*INTEG*_ yields a stable output that depends solely on the weight values of the memory, regardless of inter-chip variations. The measurement results validating this behavior are presented in section 3.2. In addition, section 2.2.1.3 discusses an offset cancelation technique for the analog circuit to enhance stability and accuracy.

##### 2.2.1.3 Offset cancelation

Since there are multiple integrator circuits within a single local cluster, offsets can influence the results of operations designed to emulate the biological neural networks. The offset cancelation methods for the comparators, integrators, and buffers in Figures 6, 7 are detailed in Behzad (2016) and Hsu and Chen (2014).

**FIGURE 8:**
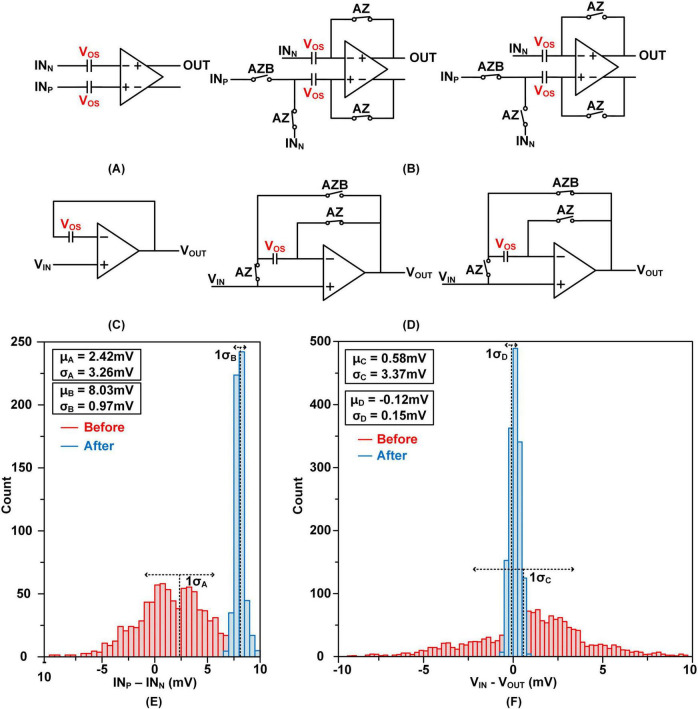
Offset Cancelation Method: **(A)** integrator and comparator without offset cancelation **(B)** integrator and comparator during offset cancelation phase and normal operation phase **(C)** buffer without offset cancelation **(D)** buffer during offset cancelation phase and normal operation phase **(E)** Monte-Carlo simulation result of the integrator and comparator and **(F)** Monte-Carlo simulation results of the buffer.

Figures 8A,C depict amplifier without offset cancelation methods and Figures 8B,D depict auto-zeroing techniques for offset cancelation. During the offset storage phase, AZ switches connect the input and output of the amplifier, allowing the offset to be stored in a capacitor. In the subsequent evaluation phase, during which the comparator, integrator, and buffer operate, AZB switches are activated to effectively cancel the stored offset.

Figure 8E shows the simulation results of the integrator and comparator. The red histogram illustrates the output before offset cancelation, with a mean difference (μ_*A*_) of 2.42 mV and a standard deviation (σ_*A*_) of 3.26 mV (approximately 10 mV at 3σ). After applying offset cancelation (blue histogram), the mean shifts to 8.03 mV (μ_*B*_), and the standard deviation (σ_*B*_) is significantly reduced to 0.97 mV, bringing the 3σ range under 3 mV. These statistical values are annotated in the figure. Figure 8F presents the buffer simulation results. Before offset cancelation, the mean and standard deviation are μ_*C*_ = 0.58 mV and σ_*C*_ = 3.37 mV, respectively. After cancelation, these are reduced to μ_*D*_ = –0.12 mV and σ_*D*_ = 0.15 mV, effectively minimizing the 3σ range to below 0.5 mV. These results demonstrate more than a 10 × improvement in error performance, achieved by circuit-level cancelation of offsets caused by process variations and parasitic mismatches.

#### 2.2.2 Integrator simulation results

**FIGURE 9:**
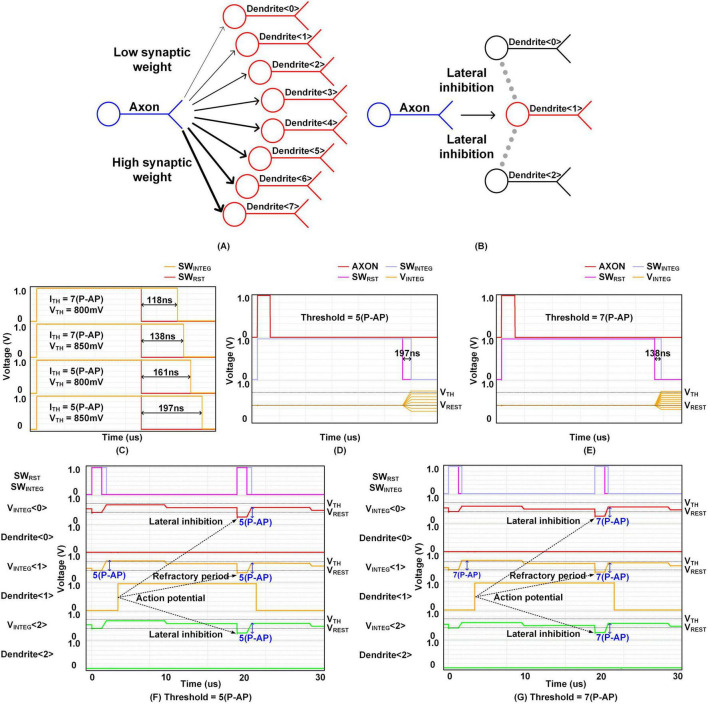
Simulation cases: **(A)** the linear integration **(B)** the relative refractory period and lateral inhibition, Simulation results: **(C)** time window generation, linear integration when threshold is **(D)** 5(P-AP) and **(E)** 7(P-AP), relative refractory period and lateral inhibition when threshold is **(F)** 5(P-AP) and **(G)** 7(P-AP).

In this section, we present the simulation results for the integrator. Figures 9A,B illustrates two specific simulation setups designed to verify the function of the proposed architecture. In both figures, the blue neuron represents the axon transmitting the stimulus, while the red neurons represent the dendrites receiving the stimulus. Figure 9A shows a scenario in which a single axon sends a stimulus to eight different dendrites, each assigned synaptic weights that differ incrementally by one level. The integration time SW_*INTEG*_ is fixed in this experiment, and the purpose is to confirm that the membrane voltage increases linearly with respect to the synaptic weight, demonstrating accurate weighted integration behavior. Figure 9B depicts a different setup, where a target dendrite receives input from an axon while neighboring dendrites are simultaneously activated to simulate lateral inhibition. This setup is used to evaluate how inhibitory activity affects the integration of excitatory input in adjacent neurons. These simulation setups were also used in actual chip measurements, and the corresponding results are presented in Figures 12, 14, respectively.

Figure 9C illustrates the signal generation process of the time window generator, as described in Figure 7. Simulations were conducted for two threshold weight configurations: 7(P-AP) and 5(P-AP), with V_*TH*_ voltage values ranging from 800 to 850 mV. The results reveal an intriguing relationship between threshold weight and integration time. As the threshold weight increases, the rate at which V_*TWG*_ rises also accelerates, leading to a shorter integration time. Conversely, higher V_*TH*_ voltage values delay the point at which V_*TWG*_ surpasses the V_*TH*_ threshold, thereby extending the integration time.

Figures 9D,E demonstrate the linear integration based on synaptic weights. When an AXON input is received, it triggers the generation of a SW_*INTEG*_ signal, and the voltage V_*INTEG*_ is obtained by converting the current through synapse columns into voltage. In the simulation result shown in Figure 9D, with a threshold of 5(P-AP) and V_*TH*_ of 850 mV, V_*INTEG*_ reflects the operation of eight synapse columns ranging from –2(P-AP) to 5(P-AP). The voltage decreases at –2(P-AP) and –1(P-AP) due to IPSPs, remains unchanged at 0(P-AP) (no weight), and increases from 1(P-AP) to 4(P-AP) due to EPSPs. At 5(P-AP), the voltage reaches the threshold, triggering an action potential. In Figure 9E, with a threshold weight of 7(P-AP) and synaptic weights ranging from –2(P-AP) to 7(P-AP), V_*INTEG*_ similarly decreases with IPSPs, remains unchanged at 0(P-AP), and increases from 1(P-AP) to 6(P-AP) due to EPSPs. The voltage surpasses the threshold at 7(P-AP), resulting in the firing of an action potential.

Figures 9F,G depict the simulation results for the relative refractory period and lateral inhibition, with threshold weights set at 5(P-AP) in Figures 9D and 7(P-AP) in Figures 9E. When the first stimulus is applied to synapses <0>, <1>, and <2>, the synaptic weight for <1> matches the threshold, causing Dendrite <1> to fire an action potential. However, the synaptic weights for <0> and <2> are below the threshold, preventing Dendrite <0> and <2> from firing action potentials. Upon the arrival of the second stimulus, V_*INTEG*_ <1> starts integration from V_*RFR*_, indicating the relative refractory period. V_*INTEG*_ <0> and V_*INTEG*_ <2> start integration from V_*LAT*_, reflecting the lateral inhibition caused by the firing of V_*INTEG*_ <1>. Even if the second stimulus matches the threshold weight, V_*INTEG*_ <1> cannot exceed the V_*TH*_ due to the refractory period. Consequently, Dendrite <0> and <2> are also unable to generate action potentials, even when their synaptic weights match the threshold, due to the effect of lateral inhibition.

## 3 Experimental results

### 3.1 Measurement setup

**FIGURE 10:**
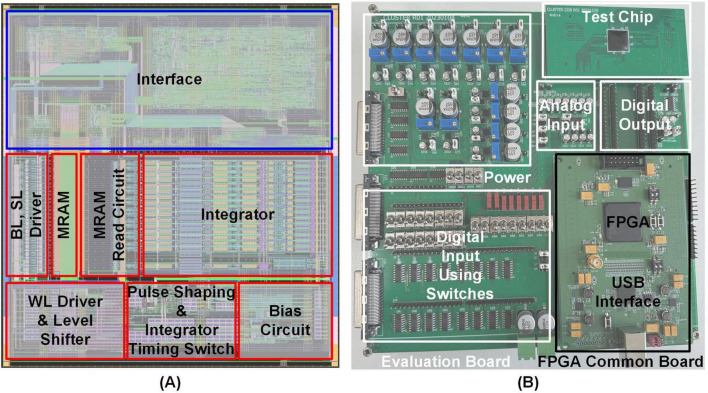
**(A)** Local cluster with interface layout **(B)** measurement setup.

Figure 10A presents the overall layout of the local cluster, designed using a 28-nm FDSOI CMOS process with MRAM. The local cluster includes an MRAM array storing synaptic weights, along with essential components such as the MRAM read circuit, WL driver, BL driver, and SL driver, all of which are crucial for retrieving values from the memory array. The local cluster also incorporates key elements such as a pulse shaping circuit, an integrator timing switch for processing management, and an integrator designed to emulate the behavior of biological neural networks.

Figure 10B shows the measurement board of the test chip, consisting of two main components: the evaluation board and the FPGA common board. The evaluation board serves multiple purposes, including hosting the test chip, supplying power via low-dropout regulators (LDOs), providing analog inputs through a digital-to-analog converter (DAC), and incorporating switches for DC inputs. It also features test pins for monitoring the outputs of the test chip. The FPGA common board facilitates digital input and output operations, featuring an I^2^C interface for communication with a PC via a USB interface. Its detachable design further enhances flexibility, allowing compatibility with other test chips and improving the overall versatility of the testing system.

### 3.2 Measurement results

**FIGURE 11:**
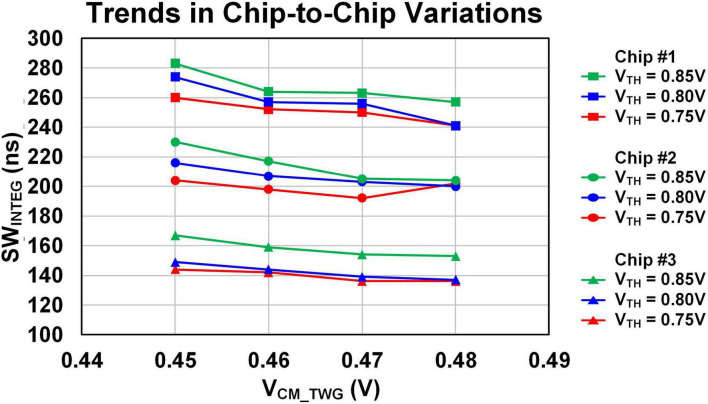
Measurement results: integration time (SW_INTEG_).

Figure 11 presents the measurement results of the integration time determined by SW_*INTEG*_, corresponding to the simulation results shown in Figure 9C. Across multiple chips with process variations, the integration time-defined as the interval between the falling edges of SW_*RST*_ and SW_*INTEG*_, as described in section 2.2.1 is proportional to the V_*TH*_ value. In other words, as V_*TH*_ increases, the integration time also increases.

As shown in Figure 11, although there is nearly a 60-ns difference among different chips due to intrinsic fabrication variation, the overall trend across the voltage range remains consistent. This demonstrates that the internally generated SW_*INTEG*_ signal effectively maintains a stable integration time based on the stored weight values in MRAM, regardless of chip-to-chip variations. The observed chip-to-chip variation is not a malfunction but rather an intended adaptive behavior of the system.

**FIGURE 12:**
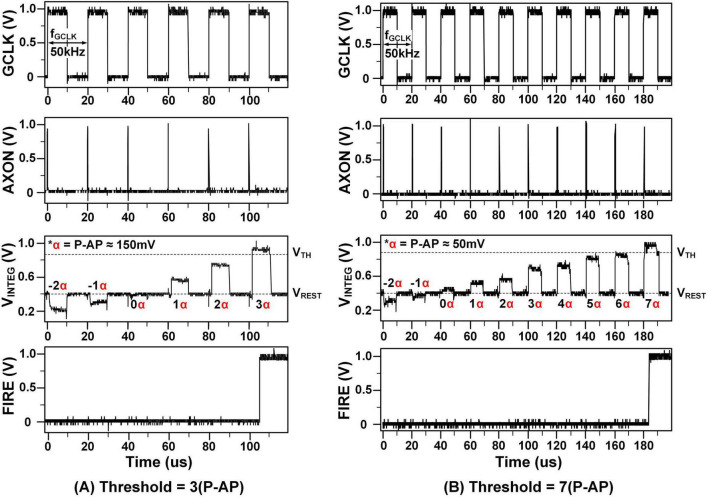
Measurement results: linear integration when threshold is **(A)** 3(P-AP) and **(B)** 7(P-AP).

The graph in Figure 12 shows the measurement results from a circuit designed to emulate the biological neural network described in section 2.1.2, confirming the linear relationship between V_*INTEG*_ values and various synapse weight values.

**FIGURE 13:**
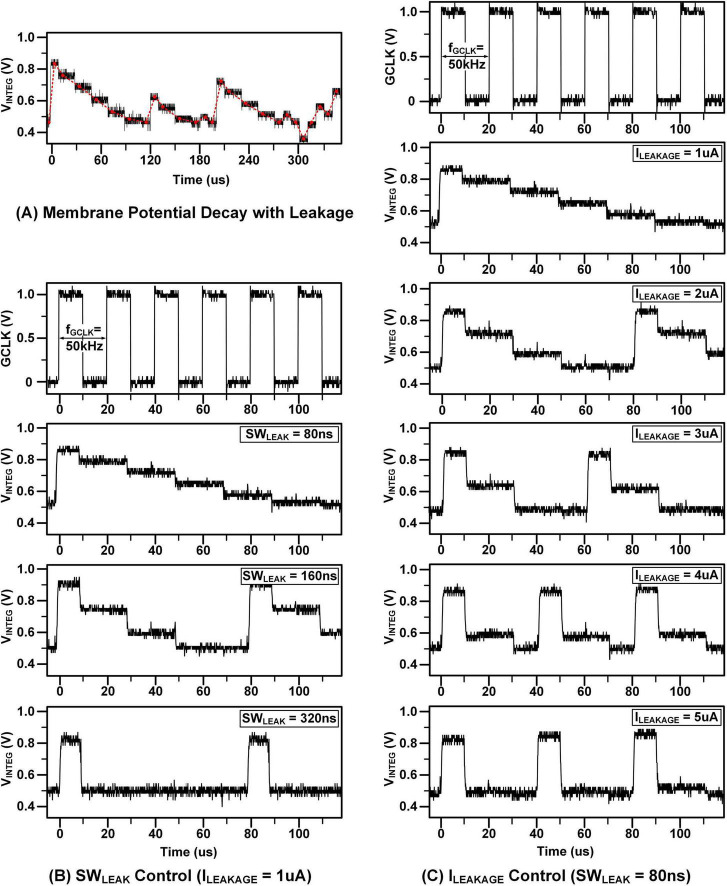
Measurement results: **(A)** measured membrane potential with leakage **(B)** leakage controlled by SW_LEAK_ **(C)** leakage controlled by I_LEAKAGE_.

The measurement results in Figures 12A,B correspond to the simulation results shown in Figures 9D,E when GCLK, which determines the frequency of AXON inputs, is set to 50 kHz. While the simulation applies stimuli with varying strengths simultaneously to multiple neurons, the measurement results were obtained by repeatedly applying and resetting input stimuli to a single neuron. Despite this difference in setup, both results consistently demonstrate linear membrane potential integration behavior. Figure 12A illustrates the case where axon inputs with synaptic weights ranging from –2(P-AP) to 3(P-AP) are received. The V_*INTEG*_ decreases when an IPSP occurs and increases when an EPSP is generated. When the synaptic weight is 3(P-AP), an action potential is fired because the weight exceeds the threshold. Figure 13B shows similar results for a threshold of 7(P-AP), with input stimuli ranging from –2(P-AP) to 7(P-AP). This result further confirms the linear integration of current into membrane voltage as a function of synaptic weight, validating the neuron’s analog processing capability across a wider dynamic range.

Figure 13A depicts the outcomes of leaky integration. As a stimulus is applied, the membrane potential undergoes integration; however, this process involves several discrete leakage steps. Unlike the continuous leakage experienced by actual biological neurons, represented by the red trend line, the proposed circuit operates with distinct leakage time windows. The leakage behavior is controlled by the SW_*LEAK*_ duration and the I_*LEAKAGE*_ current, meaning that the leakage occurs only when the SW_*LEAK*_ is activated. Figures 13B,C demonstrate the adjustable leakage mechanism. In Figure 13B, increasing the pulse width of SW_*LEAK*_ while keeping I_*LEAKAGE*_ constant leads to a greater reduction in V_*INTEG*_. In contrast, when the input current I_*LEAKAGE*_ is augmented while maintaining the pulse width of SW_*LEAK*_ constant in Figure 13C, V_*INTEG*_ experiences an even more pronounced decrease.

**FIGURE 14:**
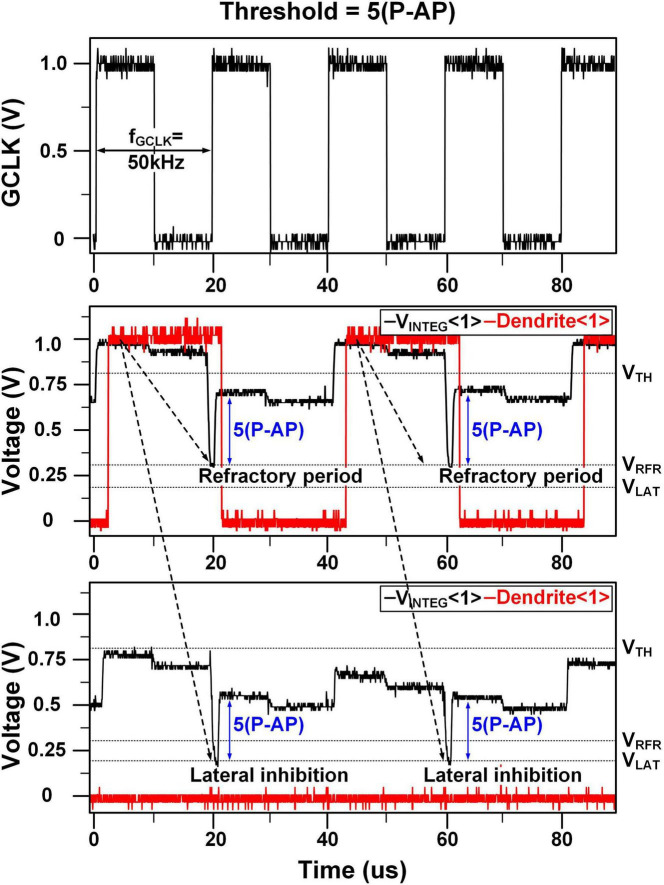
Measurement results: relative refractory period and lateral inhibition.

Figure 14 presents the measurement results demonstrating the effects of the relative refractory period and lateral inhibition, which are consistent with the simulation results shown in Figure 9F,G. When an input stimulus triggers an action potential in Dendrite <0>, the neuron enters a relative refractory period, during which the next input does not immediately lead to firing. Instead, integration resumes at a lower potential level, denoted as V_*RFR*_, upon the arrival of the next stimulus.

In contrast, Dendrite <1¿ is influenced by lateral inhibition from neighboring dendrites. Although it receives excitatory input, its membrane potential starts from a voltage significantly below the resting level due to this inhibition. As a result, while the potential rises, it does not reach the firing threshold, and no action potential is generated. Integration begins at V_*LAT*_, and the absence of spiking confirms that lateral inhibition effectively suppresses neuronal activity. This mechanism contributes to a reduced firing rate, as evidenced by the flat membrane potential trace of Dendrite <1> in Figure 14.

It is important to note that the absolute refractory period is not explicitly measured in Figure 14, as it is enforced by the circuit-level gating mechanism, which blocks incoming input stimuli during that period. This gating ensures that no new action potential can be triggered during the absolute refractory period, and therefore, no additional measurements were necessary for this phase. The behavior during the relative refractory period was sufficient to demonstrate the relevant dynamics, which are the focus of Figure 14.

### 3.3 Comparison

Table 1 provides a comparative analysis of this work with other several representative studies. A key distinguishing feature of this work is the use of STT-MRAM for the synapse array, whereas other studies employ SRAM, PCM, or ReRAM. Among these systems, our work is unique in its use of MRAM-based analog computation, which allows both excitatory and inhibitory post-synaptic potentials (EPSP/IPSP), as well as refractory period and lateral inhibition to be implemented in hardware. These biologically relevant functions are often missing or abstracted in fully digital architectures such as Burr et al. (2015), Davies et al. (2018), Ostwal et al. (2019), and Kulkarni et al. (2019).

**TABLE 1:** Comparison table.

	This Work		Loihi 2018 (Davies et al., 2018)	Neurogrid 2014 (Benjamin et al., 2014)	TED 2015 (Burr et al., 2015)	IEDM 2019 (Valentian et al., 2019)	JXCDC 2019 (Ostwal et al., 2019)	ICECS 2019 (Kulkarni et al., 2019)
*Configuration	1 Cluster	1 Chip	1 Chip	16 Chips	1 Chip	1 Chip	1 Chip	1 Chip
Technology	28 nm		14 nm FinFET	180 nm	180 nm	130 nm	–	28 nm
Memory type	STT-MRAM		SRAM	FPGA/SRAM	PCM	ReRAM	SOT-MRAM	STT-MRAM
Membrane potential	Analog		Digital	Mixed	Digital	Analog	Digital	Digital
Synapse	4k	256	128M	6G	164,885	13.5k	158,800	64k
Neuron	256	16	128k	10M	385	10	994	256
Area [mm^2^]	7.74	0.576	60	168	–	4	–	–
Neurons/mm^2^	33	27	2,184	59,523	–	2.5	–	–
**Neurons/mm^2^ [28 nm]	33	27	1,092	382,647	–	11	–	–
EPSP	O		O	O	O	O	O	O
IPSP	O		O	O	O	O	O	X
Refractory period	O		O	O	X	O	X	X
Lateral inhibition	O		O	X	X	X	X	X
Frequency range	Real-time accelerated		Real-time accelerated	Real-time	Slower than real-time	Real-time	Pulse-driven (μs level)	Accelerated

*Configuration denotes single-chip or multi-chip systems. 1 Cluster represent 16-chip implementations.

**Neurons/mm^2^ indicates that the data has been normalized. Since the studies were conducted using different processes and examined varying areas, normalization is essential to ensure a fair comparison of the data.

The table includes specifications for both a single neuromorphic chip and a 16-chip cluster. The single chip, comprising 256 synapses and 16 neurons, occupies 0.576 mm^2^, while the 16-chip cluster integrates 4,096 synapses and 256 neurons, occupying 7.74 mm^2^. For consistency with other studies in the table, the neuron count is based solely on the number of post-synaptic elements that perform LIF operations (i.e., dendrites). However, as the primary objective of this work is to emulate biologically inspired neural behavior at the circuit level, each pre-synaptic axon can also be interpreted as a distinct neuron representation, resulting in a total of 32 neurons per chip. In terms of area efficiency, although our system does not match the ultra-high neuron density of systems like Neurogrid, which heavily leverage digital scaling and simplified models, it achieves a balanced trade-off between analog biological fidelity and circuit compactness, with 33 neurons/mm^2^. Moreover, our system supports a wide frequency range, operating from 1 to 50 kHz, thus accommodating both real-time biological emulation and accelerated processing modes. In contrast, some analog systems like the ReRAM-based implementation operate in slower, pulse-driven modes, while others like (Burr et al., 2015; Ostwal et al., 2019) do not support time-continuous dynamics such as leaky integration or refractory periods. In this context, the proposed system demonstrates a practical approach to integrating MRAM with analog circuitry for implementing key neural behaviors in a compact and scalable neuromorphic architecture.

## 4 Discussion

In this paper, we have presented a biologically plausible and scalable neuromorphic system based on a mixed-signal architecture that combines MRAM-based synaptic memory with analog neuron circuits. The proposed system supports several key neural functions observed in real neurons, including leaky integration, excitatory and inhibitory postsynaptic potentials (EPSP/IPSP), refractory periods, and lateral inhibition. In addition, the system operates over a configurable temporal range (from microseconds to milliseconds), which aligns with biologically realistic firing rates and membrane time constants. This allows the circuit to replicate both behavioral and temporal dynamics of real neurons, making it suitable for experiments involving real-time interaction with biological signals. Furthermore, the global clock (GCLK) in the system is configurable from 1 kHz to 50 kHz, enabling both operation at biological time scales (e.g., 1–100 Hz) and accelerated experimental conditions, such as hardware-software co-simulation or real-time signal replay.

To realize this functionality in hardware, we chose MRAM as the synaptic memory due to its non-volatility, CMOS compatibility, and commercial availability. While MRAM offers several practical advantages, its low on/off resistance ratio poses significant challenges for analog neuromorphic computation, particularly when accurate current-mode processing is required. To address this, we introduced a current subtraction technique that enables reliable multi-level current generation from MRAM states. In addition, the circuit includes offset cancelation mechanisms and programmable current scaling via SW_INTEG_ to minimize the effects of process variation and improve the accuracy of analog computation. These mechanisms help ensure stable and consistent analog neural behavior even in the presence of device mismatch or variation of the resistance value across MRAM cells.

The system adopts a modular local cluster structure consisting of MRAM synapse arrays, analog neuron circuits, and timing control logic. By tiling multiple clusters, the network can be easily expanded without major redesigns. Interface circuits such as transmitters (TX), routers, and receivers (RX) enable efficient inter-cluster communication, supporting scalable and distributed operation. Although detailed evaluation is beyond the scope of this paper, ongoing design verification includes Network-on-Chip (NoC)-based packet transmission between clusters. Overall, this hierarchical structure lays the groundwork for expanding the architecture to larger systems.

Experimental results from fabricated test chips confirm that the proposed architecture successfully replicates key neural dynamics in hardware. Overall, this work demonstrates a practical and biologically grounded approach to implementing neuromorphic systems using MRAM-based mixed-signal circuits. Looking forward, this architecture may lend itself to integration with nano-electrode arrays (Abbott et al., 2020), potentially enabling real-time interaction with biological neural networks. Such integration could support applications in brain-inspired sensing or experimental platforms involving closed-loop biological interfacing.

## Data Availability

The original contributions presented in the study are included in the article/supplementary material, further inquiries can be directed to the corresponding author.
